# Detection of mild cognitive impairment using various types of gait tests and machine learning

**DOI:** 10.3389/fneur.2024.1354092

**Published:** 2024-07-11

**Authors:** Mahmoud Seifallahi, James E. Galvin, Behnaz Ghoraani

**Affiliations:** ^1^Department of Computer and Electrical Engineering and Computer Science, Florida Atlantic University, Boca Raton, FL, United States; ^2^Comprehensive Center for Brain Health, Department of Neurology, University of Miami, Boca Raton, FL, United States

**Keywords:** Alzheimer's disease, mild cognitive impairment, human motor behavior, gait, depth camera, machine learning, signal processing

## Abstract

**Introduction:**

Alzheimer's disease and related disorders (ADRD) progressively impair cognitive function, prompting the need for early detection to mitigate its impact. Mild Cognitive Impairment (MCI) may signal an early cognitive decline due to ADRD. Thus, developing an accessible, non-invasive method for detecting MCI is vital for initiating early interventions to prevent severe cognitive deterioration.

**Methods:**

This study explores the utility of analyzing gait patterns, a fundamental aspect of human motor behavior, on straight and oval paths for diagnosing MCI. Using a Kinect v.2 camera, we recorded the movements of 25 body joints from 25 individuals with MCI and 30 healthy older adults (HC). Signal processing, descriptive statistical analysis, and machine learning techniques were employed to analyze the skeletal gait data in both walking conditions.

**Results and discussion:**

The study demonstrated that both straight and oval walking patterns provide valuable insights for MCI detection, with a notable increase in identifiable gait features in the more complex oval walking test. The Random Forest model excelled among various algorithms, achieving an 85.50% accuracy and an 83.9% F-score in detecting MCI during oval walking tests. This research introduces a cost-effective, Kinect-based method that integrates gait analysis—a key behavioral pattern—with machine learning, offering a practical tool for MCI screening in both clinical and home environments.

## 1 Introduction

Alzheimer's disease (AD) and related dementias (ADRD) are progressive neurodegenerative diseases marked by neuronal damage and deterioration, leading to substantial cognitive impairments and affecting cognitive functions such as memory, language, and problem-solving. In addition, many individuals with ADRD have gait and balance deficits (1–4). As of 2023, approximately 6.7 million individuals in the United States aged 65 and above are estimated to live with AD, with projections indicating that this number is expected to swell to 13.8 million by 2060 (2). Despite ongoing research, a cure for ADRD remains elusive, underscoring the critical importance of early detection for managing and slowing its progression.

Mild Cognitive Impairment (MCI) is often characterized as a transitional stage of cognitive decline that exceeds the normal cognitive changes associated with aging (5) and frequently represents the earliest identifiable stage of ADRD (6). Individuals with MCI due to AD exhibit AD pathology biomarkers and face a significant risk of transitioning to clinical AD dementia, with an annual progression rate ranging from 10% to 15% (7). Furthermore, MCI is also prevalent among patients with Parkinson's disease (PD), representing a significant subset of this population that may transition to PD dementia. Recent studies have begun to explore biomarkers that differentiate between PD patients with and without MCI, enriching the understanding and potential intervention strategies for these conditions (8). MCI may also represent early stages of vascular cognitive impairment, and in some cases may not progress to dementia.

In clinical settings, current diagnostic procedures involve a collection of tests, including magnetic resonance imaging, positron emission tomography, lumbar puncture, blood tests, and neuropsychological evaluations, which can provide comprehensive information for the diagnosis of MCI and ADRD among older adults (9, 10). Although these clinical tools offer comprehensive insights into the cognitive status and underlying causes of impairment in older adults, they are expensive, invasive, and time-consuming, requiring clinical expertise (9) and less frequently used in the primary care setting. The diagnosis process also has a significant subjective component and depends on the knowledge and experience of the clinician or researcher. Recent reports support that detection of early stages of ADRD and MCI is challenging outside of specialty centers, with most cases recognized in the primary care settings at the moderate stage of impairment and nearly 80% of MCI cases not diagnosed at all (11, 12). A comprehensive neurological and neuropsychological evaluation is the standard in memory care centers but are less common in primary care. Thus, developing new technological tools with lower cost, easy-to-set, and objective decisions can increase the likelihood of early detection of MCI and AD in the primary care setting. This has advantages for health disparity populations, who may not have access to specialists or expensive technology.

Gait and balance assessments are commonly examined in the primary care settings and are emerging as promising tools in this context for MCI and ADRD detection (13). Gait, a fundamental human function, is integral to daily life activities and involves complex cognitive processes (14). As a routine activity, gait necessitates the integration of attention, planning, memory, and various motor and perceptual functions (15). This intricate interplay between cognitive and motor functions makes gait analysis a potentially valuable behavioral marker for early detection of cognitive decline. By focusing on gait patterns, we can tap into these underlying cognitive processes, offering a non-invasive and insightful window into the cognitive health of older adults.

Previous studies have primarily concentrated on exploring the association between gait features and MCI or textcolorblackADRD, aiming to identify potential biomarkers for their detection (16, 17). These studies typically employed electronic walkways, wearable sensors, or systems comprising multiple infrared cameras with reflective markers attached to participants' bodies for gait recording (17, 18). However, their analyses were confined mainly to descriptive statistical evaluations of gait data to identify possible biomarkers for MCI or AD. Such gait recording systems, often requiring specialized setup and being costly, are generally limited to clinical environments. During gait tests, they also tend to overlook the tracking and recording of movements across various joints and limbs, which could yield more comprehensive insights and novel biomarkers for MCI and ADRD detection.

The majority of these clinical studies have focused on straight walking, primarily due to the limitations of their recording systems, such as computerized pressure mats, or have restricted their analyses to the Timed Up and Go test (TUG), which includes only brief turning sequences (19, 20). While a few studies have ventured into developing machine learning methods to detect MCI or ADRD using straight walking data captured with these recording systems (21, 22), they have not extensively explored gait analysis in varied conditions like oval and straight walking paths using non-wearable technology. Such an approach could potentially eliminate the influence of recording systems on the natural gait pattern of participants.

Furthermore, a comprehensive analysis comparing machine learning techniques to objectively assess older adults' cognitive status through straight and oval-path walking remains unexplored. While gait analysis combined with machine learning shows promise as a tool for detecting MCI in older adults, there is a gap in research regarding the application of non-wearable technologies in diverse walking conditions and the comparative evaluation of machine learning methods in this context.

This study introduces a novel and substantive advancement in gait analysis by developing a new system utilizing the Kinect v.2 camera. Traditional methods for gait analysis often rely on computerized pressure mats, which are long force plates allowing a person to walk as they measure gait, multicamera video-based motion capture systems combined with markers mounted on the body, and wearable sensors (23–25). Each of these technologies provides valuable quantitative measurements of gait and balance, but they also have limitations. Computerized pressure mats can provide precise measurements but are costly and require dedicated space, limiting their accessibility and feasibility in various settings. Wearable sensors present challenges in consistent device placement and calibration and may not reliably capture comprehensive gait markers such as spatial parameters like step width. Marker-based camera systems (multicamera video-based) require the placement of multiple cameras and a collection of reflective markers on the body, which can be cumbersome and invasive for participants. Additionally, synchronizing multiple cameras and the overall cost of such systems pose significant challenges. Given these challenges, depth cameras offer significant potential for an accessible and comprehensive movement assessment. Kinect v.2 depth camera, a non-invasive and non-wearable technology developed by Microsoft Corporation, can track 25 body joints more than conventional gait recording systems (see Supplementary Table S1 for detailed features). It employs Time of Flight (ToF) technology for depth measurement, offering enhanced performance, accuracy, and a broader field of view. The Kinect v.2 is particularly effective within a measurement range of 0.5 to 4.5 meters. It can simultaneously detect and track up to six individuals, making it highly suitable for detailed gait analysis in varied settings. It also offers the dual advantage of not influencing natural gait patterns during testing and addressing privacy concerns by relying solely on joint movement data without needing actual image data. This makes our system less costly and more accessible, making it suitable for clinical and non-clinical environments.

Our proposed system's contributions are multifold. We extract a comprehensive set of gait features using recorded signals of 25 main body joints detected and tracked by Kinect v.2 camera, including both macro and micro-level details, to provide richer insights into the gait of older adults. By comparing gait patterns in straight and oval-path walking, our approach enhances the understanding of the sensitivity of different gait tests in detecting MCI. The integration of signal processing, descriptive statistical analysis, and machine learning in our methodology not only differentiates healthy older adults and those with MCI but also increases the accuracy and sensitivity of the diagnosis. Further, our system optimizes the detection process by focusing on a smaller yet more powerful set of unique gait features for MCI detection, facilitating faster and more efficient diagnosis. Ultimately, this novel system holds the potential to significantly increase the early detection of cognitive impairment in older adults at the MCI stage, potentially preventing progression to ADRD.

## 2 Materials and methods

### 2.1 Participants

A total of 55 adults 60 years or older, including 30 healthy control (HC) without any cognitive impairment and 25 older adults with MCI, were enrolled in the present study. The MCI participants were the clients of Iran Dementia and Alzheimer's Association (IDAA) who underwent a comprehensive diagnosis process including neuropsychological tests, Magnetic Resonance Imaging (MRI), and EEG by a medical expert board of that center. The HC group consisted of the clients of the IDAA who visited the center for regular checkups, participated in prevention programs held by the IDAA, and were community volunteers. Of the HC participants, 17 were IDAA clients, and 13 were community volunteers. The HC group also underwent various checkups and diagnosis protocols by the center's medical expert board. The participants noticed our study using the IDAA center or responded to notices and announcements about the study being published in different communities.

The inclusion criteria for the participants were people who could perform the gait tests independently. Also, they had no stroke or knee or hip displacements, which could affect their common gait patterns. The people who have severe depression were excluded from the study, and the depression level was measured by the Persian version of the Geriatric Depression Scale (GDS). Further, the Persian version of the Mini-Mental State Examination (MMSE) and Montreal Cognitive Assessment (MoCA) were used for cognitive screening of participants. The demographic and clinical information of the participants in this study who completed all the gait tests is presented in Table 1. The ethics committee of Semnan University of Medical Sciences of Iran confirmed the study under Protocol No. IR.SEMUMS.REC.1398.237, date of approval 2019.12.17, and performed in line with the Declaration of Helsinki. Before participating in the study, comprehensive information about the study was presented to the volunteers, and they could leave the study at any stage they wanted.

**Table 1:** Demographic and clinical information of participants.

**Characteristic**	**HC (*****N*** **= 30)**	**MCI (*****N*** **= 25)**	***p*** **-value**
Age (years)	68.33 ± 2.15	69.76 ± 6.45	0.091
Female gender, N (%)	18 (60)	19 (76)	0.216
BMI (kg/*m*^2^)	24.51 ± 2.67	26.67 ± 2.62	<0.001^*^
Years of education	13.53 ± 3.05	11.56 ± 3.00	0.008^*^
MMSE	28.50 ± 1.17	25.60 ± 1.29	<0.001^*^
MoCA	27.13 ± 2.05	22.76 ± 1.69	<0.001^*^
GDS	1.43 ± 1.33	3.52 ± 1.29	<0.001^*^

Mean ± standard deviation was shown. *N*, Number of participants; HC, Healthy Cognitive Control Group; MCI, Mild Cognitive Impairment; BMI, Body Mass Index; MMSE, Mini-Mental State Examination (maximum score, 30); MoCA, Montreal Cognitive Assessment (maximum score, 30); GDS, Geriatric Depression Scale (maximum score, 15). The gait feature analysis was adjusted for the confounding variables of BMI, education, and GDS.

* Significant difference at *p*-value < 0.05.

### 2.2 Gait measurements

The 10-meter-walking test, a standard gait test at clinics (26), was recorded from the participants in the single-cognitive task condition for straight and oval paths. Supplementary Section S2 provides details of the oval path. The participants were given instructions before performing these gait tests, and they could practice the test three times before the trial recording process. The participants walked at their preferred gait speed without support from the recording and healthcare team and did the gait tests independently. They rested up to 5 min between the recordings as they needed. Participants selected their preferred direction–clockwise or counterclockwise–for the oval walking test, enhancing the authenticity of gait data by accommodating natural walking tendencies and reducing potential performance anxiety.

A single Kinect v.2 camera, a depth camera from Microsoft Corporation, was connected to an ASUS-FX503 laptop with Intel Core i7-7700HQ CPU@2.80 GHz 2.80 GHz processor and 8.00GB of Installed Memory (RAM). The Kinect camera was mounted on a tripod next to the recording paths, and the recording process was controlled by a Graphical User Interface (GUI) developed in MATLAB 2019. We have included detailed information about the open-source tools and software packages used in our research to record and process data in the Supplementary Section S3. The Kinect v.2 camera can detect and track 25 joints of the participant's body and record the RGB and depth data (27, 28). The accuracy of pose estimation with Kinect v2 is influenced by the type of activity, camera setup, and environmental factors. Supplementary Section S4 details our data collection methodology and the reliability of our analysis, emphasizing how we carefully controlled environmental variables and optimized camera settings to ensure reliable data collection in a clinical research setting.

**Figure 1:**
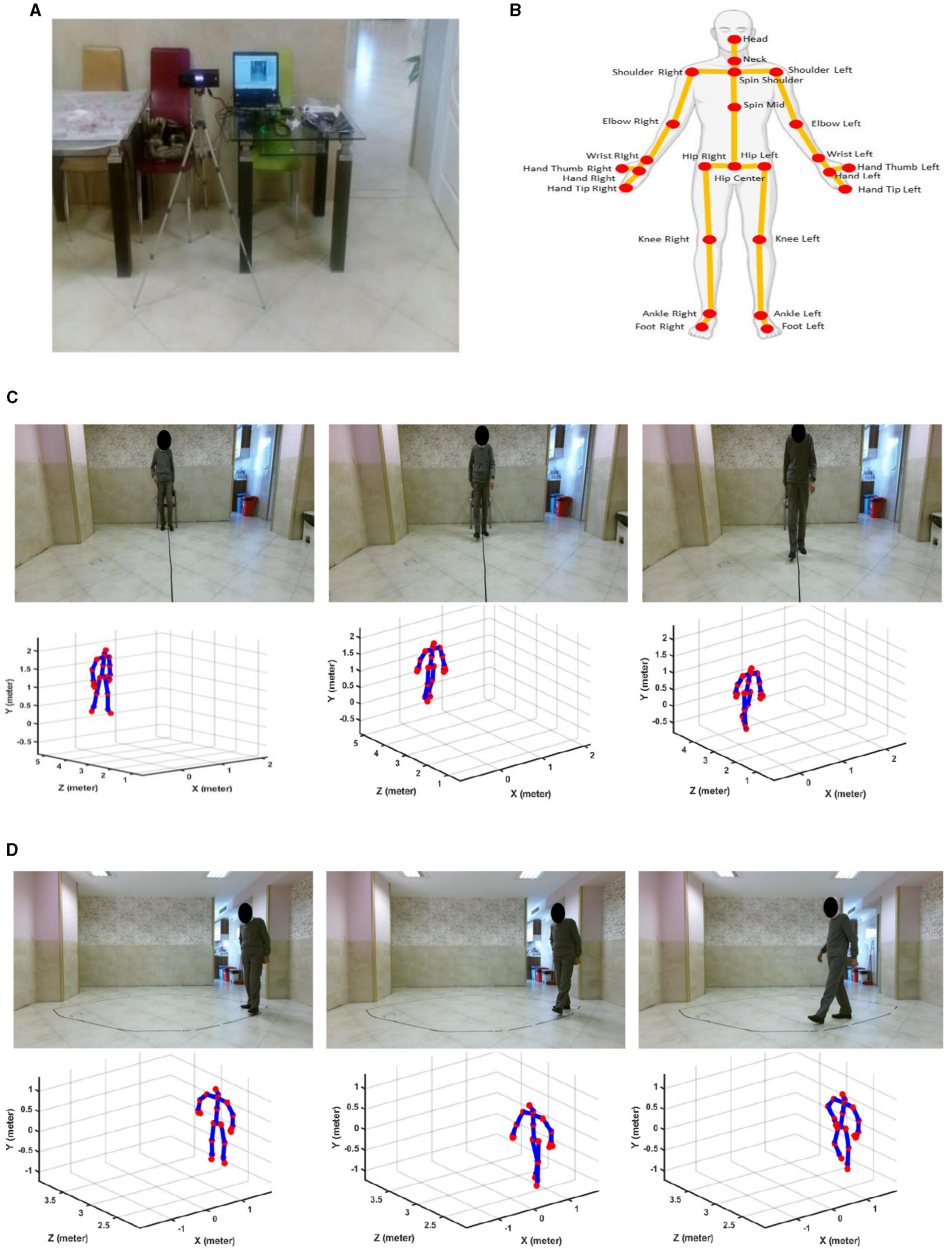
Recording tools and sample data. **(A)** Kinect v.2 camera connected to a laptop. **(B)** Trackable joints of the body via Kinect v.2 camera. **(C)** RGB and skeletal data for straight path walking. **(D)** RGB and skeletal data for oval path walking.

Figure 1 shows the recording tools and settings, and 25 of the body's joints can be detected and tracked by a Kinect v.2 camera. In this study, we used only the skeletal data, which are the signals of movements of the body joints detected and tracked by the Kinect v.2 camera for further gait analysis. We used the skeletal data because this type of data can provide comprehensive information about the movement of 25 main body's joints, as seen in Figure 1B, while the privacy issue and the high-cost hardware for the following analysis are solved. Figures 1C, D show the samples of recorded RGB and skeletal data from a participant while performing straight and oval walking tests, respectively. Supplementary Section S5 provides more detail for recorded straight and oval walking data, confirming the acceptable range of error for tracking the subject's body joints during these tests.

As shown in Figure 1, the camera was placed frontally for straight-path walking to ensure clear, unobstructed data capture and laterally for oval-path walking to manage space constraints and minimize self-occlusion issues associated with lateral movements. It is important to note that healthy controls and MCI participants underwent gait analysis under identical camera setups for straight and oval path walking tests. This ensured that any differences observed in detecting MCI were due to genuine gait variations and not influenced by differences in camera placement. Additional analysis was conducted and detailed in Supplementary Section S6. This analysis examined the effect of camera view changes on estimating ankle-foot joint distances. The results showed no significant difference in joint distance measurements between the camera views, confirming that the observed differences in gait features between HC and MCI participants are not attributable to camera placement biases.

### 2.3 Data processing

**Figure 2:**
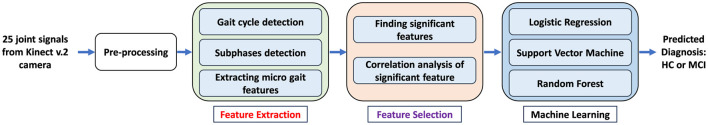
General steps of the proposed algorithm for processing and analysis of recorded skeletal data for gait tests to detect MCI.

After recording data, a comprehensive analysis was done on the skeletal data using signal processing algorithms, descriptive statistical analysis, and machine learning tools. These processes included prepossessing, feature extraction, feature selection, and participant classification using different machine learning algorithms. Figure 2 shows the general steps of the proposed algorithm for data processing of the recorded data, which are described in more detail in the following.

#### 2.3.1 Preprocessing

The location changes of the 25 body's joints, which were detected and tracked by Kinect v.2 camera for gait tests, can be presented as the signals during time. In the preprocessing step, we applied a six-order Butterworth filter with a cut-off frequency of 3 Hz to remove noises from the movement signals of the body's joints (29, 30).

#### 2.3.2 Feature extraction

We extracted a comprehensive collection of 50 gait features (see Table 2) from the preprocessed signals of the body's joints for separate straight and oval path walking tests. The feature extraction algorithms included several steps. For macro features like average velocity, which shows the general performance of the participant for the gait test, we used the total displacement of the foot joint from the starting to the end point of the walking tests, and it was divided by the duration needed to finish the test (31). However, for 49 remaining extracted features, we needed to detect the gait cycles and their subphases like step, stance, and swing phases, then calculate the features like step time, step length, swing time, etc. These types of features are usually called micro features as they can provide more detail about the gait performance of older adults.

**Table 2:** Extracted features from straight and oval walking and their comparison results.

**Feature**		**Straight walking**			**Oval walking**		
**Type**	**Name**	**HC**	**MCI**	***p*-value**	**HC**	**MCI**	***p*** **-value**
Macro	Velocity (cm/s)	50.13 ± 27.92	47.39 ± 9.80	0.780	45.90 ± 8.89	39.27 ± 10.09	0.007^*^
	Cadence (steps/minute)	68.22 ± 10.55	68.90 ± 9.70	0.247	62.02 ± 9.73	55.00 ± 11.67	0.018^*^
Micro temporal	Stance T. me (s)	0.78 ± 0.24	0.83 ± 0.34	0.716	0.57 ± 0.15	0.64 ± 0.22	0.138
	Stance T. var (%)	18.15 ± 20.91	23.48 ± 26.30	0.993	49.91 ± 18.75	73.08 ± 20.27	<0.001^*^
	Stance T. med (s)	0.78 ± 0.24	0.84 ± 0.33	0.729	0.53 ± 0.17	0.52 ± 0.23	0.384
	Swing T. me (s)	0.71 ± 0.15	0.72 ± 0.16	0.841	0.54 ± 0.04	0.52 ± 0.07	0.211
	Swing T. var (%)	12.41 ± 16.09	19.83 ± 13.89	0.053	26.10 ± 8.00	32.89 ± 11.12	0.018^*^
	Swing T. med (s)	0.69 ± 0.15	0.72 ± 0.16	0.742	0.51 ± 0.05	0.48 ± 0.06	0.001^*^
	DS T. me (s)	0.21 ± 0.11	0.25 ± 0.14	0.323	0.21 ± 0.04	0.31 ± 0.09	<0.001^*^
	DS T. var (%)	69.63 ± 38.82	80.65 ± 26.45	0.407	80.16 ± 22.57	91.91 ± 21.54	0.072
	DS T. med (s)	0.13 ± 0.08	0.17 ± 0.10	0.080	0.17 ± 0.05	0.23 ± 0.05	<0.001^*^
	SS T. me (s)	0.53 ± 0.16	0.52 ± 0.15	0.498	0.40 ± 0.08	0.35 ± 0.09	0.014^*^
	SS T. var (%)	37.30 ± 23.06	45.14 ± 25.72	0.196	49.00 ± 13.07	48.56 ± 11.29	0.896
	SS T. med (s)	0.57 ± 0.18	0.55 ± 0.18	0.496	0.36 ± 0.09	0.34 ± 0.10	0.158
	Step T. me (s)	0.77 ± 0.13	0.78 ± 0.11	0.331	0.88 ± 0.13	1.05 ± 0.27	0.012^*^
	Step T. var (%)	18.05 ± 16.60	27.13 ± 11.29	0.042^*^	34.08 ± 17.07	40.51 ± 10.50	0.017^*^
	Step T. med (s)	0.76 ± 0.09	0.77 ± 0.10	0.606	0.83 ± 0.09	0.94 ± 0.20	0.013^*^
	Step T. sym me	0.64 ± 0.08	0.64 ± 0.06	0.680	0.83 ± 0.03	0.84 ± 0.03	0.207
	Step T. sym var(%)	24.22 ± 20.21	34.77 ± 14.35	0.035^*^	14.64 ± 5.93	14.45 ± 5.27	0.899
	Step T. sym med	0.68 ± 0.08	0.70 ± 0.08	0.452	0.87 ± 0.03	0.88 ± 0.03	0.019^*^
	Stride T. me (s)	1.55 ± 0.30	1.54 ± 0.22	0.254	1.77 ± 0.26	2.08 ± 0.53	0.016^*^
	Stride T. var (%)	13.66 ± 14.58	19.61 ± 9.54	0.049^*^	26.12 ± 12.36	29.41 ± 10.98	0.261
	Stride T. med (s)	1.57 ± 0.30	1.58 ± 0.25	0.496	1.67 ± 0.18	1.92 ± 0.42	0.024^*^
	Stride T.reg me	0.94 ± 0.06	0.77 ± 0.12	<0.001^*^	0.76 ± 0.09	0.74 ± 0.09	0.414
	Stride T. reg var (%)	0.37 ± 2.35	5.73 ± 10.14	0.118	21.75 ± 14.39	18.65 ± 9.16	0.729
	Stride T. reg med	0.94 ± 0.06	0.77 ± 0.12	<0.001^*^	0.75 ± 0.11	0.74 ± 0.11	0.676
Micro spatial	Step L. me (cm)	35.75 ± 5.53	31.06 ± 6.74	0.003^*^	37.58 ± 4.97	32.91 ± 3.46	<0.001^*^
	Step L. var (%)	21.21 ± 18.29	32.65 ± 19.51	0.016^*^	37.01 ± 9.97	41.84 ± 9.57	0.075
	Step L. med (cm)	37.63 ± 5.12	30.99 ± 9.19	<0.001^*^	40.16 ± 5.76	34.22 ± 4.90	<0.001^*^
	Step L. sym me	0.80 ± 0.17	0.74 ± 0.19	0.106	0.70 ± 0.10	0.66 ± 0.11	0.114
	Step L. sym var (%)	22.16 ± 31.41	21.88 ± 15.86	0.174	34.87 ± 12.43	38.87 ± 13.25	0.255
	Step L. sym med	0.83 ± 0.18	0.75 ± 0.19	0.025^*^	0.75 ± 0.13	0.67 ± 0.16	0.030^*^
	Step W. me (m)	0.12 ± 0.03	0.13 ± 0.03	0.919	0.21 ± 0.04	0.17 ± 0.03	0.002^*^
	Step W. var (%)	30.91 ± 12.54	23.10 ± 11.13	0.013^*^	56.82 ± 12.33	67.09 ± 13.87	0.011^*^
	Step W. med (m)	0.13 ± 0.03	0.13 ± 0.03	0.912	0.21 ± 0.06	0.16 ± 0.04	0.022^*^
	Step H. me (m)	0.11 ± 0.04	0.08 ± 0.04	0.003^*^	0.12 ± 0.02	0.13 ± 0.02	0.069
	Step H. var (%)	28.56 ± 12.18	39.24 ± 14.15	0.012^*^	38.65 ± 14.60	46.29 ± 11.20	0.004^*^
	Step H. med (m)	0.11 ± 0.04	0.08 ± 0.05	0.020^*^	0.11 ± 0.02	0.12 ± 0.02	0.114
	Stride L. me (cm)	74.02 ± 11.16	63.62 ± 14.92	0.006^*^	75.84 ± 10.44	65.91 ± 6.83	<0.001^*^
	Stride L. var (%)	11.82 ± 14.75	21.45 ± 13.08	0.013^*^	27.84 ± 11.09	30.83 ± 12.33	0.393
	Stride L. med (cm)	74.36 ± 12.32	63.88 ± 15.35	0.002^*^	76.46 ± 11.61	65.10 ± 9.50	<0.001^*^
	Stride L. reg me	0.96 ± 0.06	0.79 ± 0.13	<0.001^*^	0.68 ± 0.12	0.70 ± 0.12	0.657
	Stride L. reg var (%)	2.44 ± 2.72	6.60 ± 14.42	1.00	23.37 ± 15.17	24.66 ± 14.71	0.666
	Stride L. reg med	0.96 ± 0.06	0.79 ± 0.13	<0.001^*^	0.68 ± 0.13	0.71 ± 0.15	0.369
Micro spatiotemporal	Step V. me (cm/s)	47.26 ± 7.04	45.62 ± 13.01	0.574	46.41 ± 8.05	37.17 ± 8.08	<0.001^*^
	Step V. var (%)	30.75 ± 19.45	52.01 ± 21.87	<0.001^*^	47.66 ± 9.92	53.47 ± 14.37	0.083
	Step V. med (cm/s)	47.65 ± 8.28	41.67 ± 12.88	0.139	46.88 ± 9.48	35.08 ± 9.92	<0.001^*^
	Stride V. me (cm/s)	48.08 ± 7.22	43.65 ± 12.81	0.177	45.51 ± 7.91	35.47 ± 8.51	<0.001^*^
	Stride V. var (%)	14.47 ± 14.88	28.80 ± 13.21	0.001^*^	37.78 ± 11.52	38.89 ± 10.05	0.704
	Stride V. med (cm/s)	48.13 ± 7.97	43.16 ± 12.73	0.165	46.35 ± 8.48	34.15 ± 10.06	<0.001^*^

Data are shown as mean ± standard deviation; *p*-value is reported for comparison between two study groups;

* Significant difference at *p*-value < 0.05.

HC, healthy control without cognitive impairment; MCI, mild cognitive impairment; T, time; L, length; W, width; H, height; V, velocity; me, mean; var, variability; sym, symmetry; reg, regularity; DS, double support; SS, single support.

**Figure 3:**
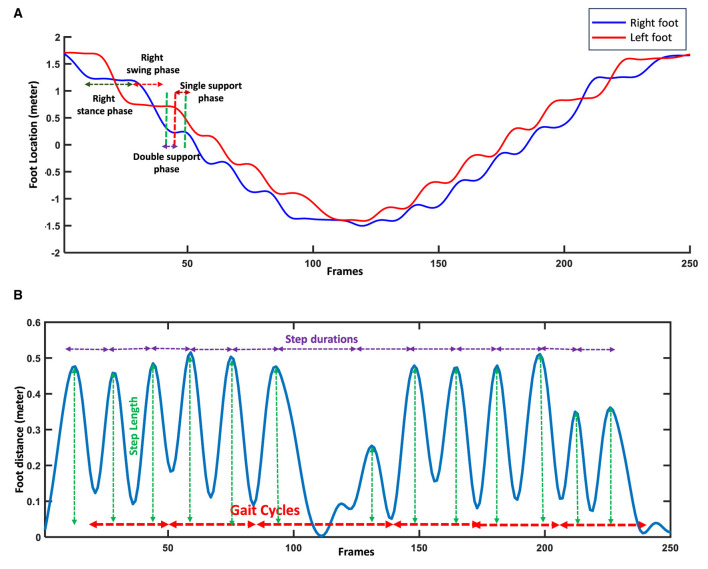
Feature extraction using right and left foot signals and their distance. **(A)** Detecting the subphases of gait cycles using the stability or changes in the locations of feet. **(B)** Detecting gait cycles and steps using distance signal of feet.

Several analyses were done on the right and left foot joints to extract the micro features. Figure 3 shows the plotted signals of the right and left foot and their distance signal for one of the participants while performing the gait test. First, we detected the gait cycle by plotting the distance signals of the right and left feet and their peaks. Each gait cycle is defined as the duration a foot contacts the ground to when the same foot again contacts the ground (32). Each gait cycle comprises two successive steps; two successive steps are also known as stride or a single gait cycle. Thus, we created the distance signal of the right and left feet and then used the duration between two successive peaks or valleys of this signal to find the gait cycles (Figure 3B). Each peak of the distance signal of the feet shows the steps' location and length (Figure 3B). To extract the features from the subphases of the gait cycles, including the stance time, swing time, single support time, and double support time, the derivative of the right and left foot signals were used (31). For the stance subphase of one leg, the foot has no location changes and remains in contact with the ground (Figure 3A). Thus, its derivative of the foot signal is approximately zeros. In comparison, for the swing phase of a foot, the derivative of the movement signal is not zero because the foot's location changes. For the subphases of single support, the location of one of the feet changes, and the other foot does not change, while in the double support phase, both of the feet locations do not change (Figure 3A). Thus, in the single support, the derivative of one foot is zero, and the other is non-zero, while in the double support phase, the derivative of signals for both feet' movement are zeros. Figure 3A shows the gait cycles and their subphases extracted from the signals of the body's joints during walking. After detecting the various subphases of gait cycles, statistical metrics, including the mean, median, and variability of the extracted subphases, were calculated to provide information about the performance of the participants during the whole process of gait tests. Various statistical metrics were calculated for micro gait features consisting of mean, median, variability, symmetry, or regularity. A feature's variability is calculated by dividing the standard deviation of a feature by its mean, which can be presented using the percentage (33). Equation 1 shows the variability of micro features.


(1) $$\begin{array}{c} var\left(x\right)=\frac{std\left(x\right)}{mean\left(x\right)}*100 \end{array}$$

*var(x)* and *std* show the variability and standard deviation of a micro feature of x, respectively.

The symmetry and regularity of step and stride features show the similarity between the right and left feet during walking. Equation 2 shows this similarity index (34, 35).


(2) $$\begin{array}{c} SI\left(x\right)=1-\frac{\left|x_{right}-x_{left}\right|}{max\left(x_{right},x_{left}\right)} \end{array}$$

SI(x) shows the index of symmetry or regularity of features x for step or stride during walking. *x*_*right*_ and *x*_*left*_ are the values of the gait features for right and left feet, respectively. The range of this index can change from 0 to 1. The higher value of SI(x) means higher symmetry or regularity for the x feature.

#### 2.3.3 Feature selection

Before determining the significant features, we investigated the data for any confounding factors. The demographic and clinical information of the participants, including age, Body Mass Index (BMI), years of education, and neuropsychological test scores, were compared between two study groups using descriptive statistical analysis methods. Shapiro-Wilk was used for normality check (36). Unpaired t-test and Mann-Whitney U tests were applied for normally distributed and non-normally distributed demographic and clinical information, which were numerical variables (37). For categorical variables like gender, the chi-square test was used (38). Our analysis showed significant differences between the two study groups for confounding variables of BMI, years of education, and GDS scores, which can affect the gait tests. Thus, we adjusted the extracted gait features using ANCOVA (Analysis of Covariance) and removed the effect of those confounding variables (39).

We conducted feature selection separately for straight walking gait markers and oval path markers to distinguish between MCI and HC participants. We employed two strategies: traditional statistical methods commonly used in clinical research and machine learning techniques.

Statistical approach: Our process involves a two-step method. Initially, we evaluate each gait feature for statistical significance. The normality of each feature is assessed using the Shapiro-Wilk test. Features conforming to a normal distribution are analyzed with the unpaired *t*-test. At the same time, those who do not meet this criterion are evaluated using the Mann-Whitney U test, with a significance threshold set at *p* < 0.05. This first phase identifies features with significant differences between the study groups. We conduct a correlation analysis among the significant features in the second phase. For any set of features demonstrating a correlation coefficient greater than 90%, we select the feature with the lowest *p*-value. This strategy reduces redundancy and concentrates on the most discriminative features, thereby aiding clinical experts by highlighting essential gait biomarkers for MCI detection. This refined set of features also simplifies the input for classification methods, potentially enhancing the accuracy and efficiency of tools like Logistic Regression (LR) and Support Vector Machine (SVM).

Machine learning approach: we also utilized a Random Forest (RF) algorithm, detailed further in Section 2.3.4. The RF was applied to all extracted features from the oval and straight-walking datasets without preliminary selection. This approach allows the RF model itself to determine the importance of each feature, offering an unbiased insight into which features most effectively differentiate between MCI and HC.

#### 2.3.4 Machine learning methods

In this study, we applied three different machine learning models to discriminate the MCI and HC participants using selected gait features from straight and oval path walking tests separately. Our three classifiers were LR, SVM, and RF to classify participants into MCI and HC. We chose these different types of classifiers because each of them uses a different method for finding the decision boundary between two study groups.

##### 2.3.4.1 Logistic regression

LR is a straightforward and highly effective classifier for binary and linear classification challenges (40). Despite its simplicity, this classifier demonstrates remarkable efficacy in addressing binary problems and is frequently employed in medical and clinical investigations (41). LR is a transformation of a linear regression using a sigmoid function. The input of the logistic function is the vector of features, while its output is the output of a Sigmoid function ranging from 0 to 1 (42). Equation 3 shows the formula of the LR classifier.


(3) $$\begin{array}{c} f\left(X\right)=\frac{1}{1+e^{w_{0}+w_{1}x_{1}+...+w_{n}x_{n}}} \end{array}$$

*X* = *x*_1_, ..., *x*_*n*_ shows the vectors of the input features, and the *f*(*X*) shows the output of the sigmoid function. *W* = *w*_1_, ..., *w*_*n*_ are the weights or parameters of the LR classifier, which are optimized using the training and validation data to fit the generalized models on the data and then applied to the new test data.

##### 2.3.4.2 Support vector machine

SVM represents a supervised binary classifier commonly advocated for the analysis of clinical data, particularly when the dimensionality of the features exceeds the number of available samples (43). This classifier endeavors to distinguish between two study groups by delineating a linear hyperplane, and in cases where the data is not linearly separable, a transformation into a new space is achieved using kernels (44). Equation 4 shows mapping samples of *X*_*i*_ and *X*_*j*_ to a new feature space using the map ϕ.


(4) $$\begin{array}{c} K\left(X_{i},X_{j}\right)=\left(\Phi \left(X_{i}\right).\Phi \left(X_{j}\right)\right) \end{array}$$

K is the kernel for mapping of *X*_*i*_ and *X*_*j*_ samples. To simplify the mapping process, particular kernels like linear, polynomial, or Radial Basis Function (RBF) are usually used in practical problems. After mapping features to a space where the data can be separated linearly, the optimal weights for a separable hyperplane with the maximum margin are found using the training data. Equation 5 shows the final nonlinear decision function for the SVM classifier.


(5) $$\begin{array}{c} f\left(X\right)=sign\left(\overset{n}{\underset{i=1}{\sum }}W_{i}.K\left(X,X_{i}\right)+b\right) \end{array}$$

f(X) shows the decision (label) predicted for the sample of X. n is the dimension of the features for each sample of data. K is the kernel for mapping the data. *W* = *w*_1_, ..., *w*_*n*_ are the weights (coefficients) of the decision hyperplane, and b is the intercept. These hyperplane parameters are found using the training data to maximize the distance between the hyperplane and the training samples. The optimal hyperplane is called the maximal margin hyperplane and is used to make decisions about the test data.

The optimization of the SVM model for subsequent applications to test data involves the adjustment of critical parameters, such as the selection of the kernel type, its associated parameters, and regularization, all of which are fine-tuned utilizing the training and validation data in the training step.

##### 2.3.4.3 Random forest

RF is a classifier that combines the output of several decision trees to achieve a single final decision (45). This algorithm is an extension of the bagging method, which uses bagging and feature randomness to create an uncorrelated forest of decision trees (46). Given a training set *D* = *X*_1_, ..., *X*_*m*_ with the labels *Y* = *y*_1_, ...., *y*_*m*_, bagging selects a random subset of training data for *b* = 1, ..., *B* (B times) to make different trees. The results of the B trees are combined with the majority voting for classification and averaging for the regression problem to predict the output for the test sample. Considering *f*_*b*_ as a decision tree classifier and Ensemble *E* = *f*_1_, ..., *f*_*B*_ is the collection of classifiers. The decision of the *b*^*th*^ classifier (*f*_*b*_) is denoted by *d*_*b,j*_ ∈ {0, 1} while *j* = 1, 2, ..., *Q* and k is the number of classes. The decision tree of *f*_*b*_ will produce *d*_*b,j*_ = 1 if that classifier predicts a class or label of *j*, and is *d*_*b,j*_ = 0 otherwise. The final decision about sample X using majority voting of B classifiers can be shown by Equation 6.


(6) $$\begin{array}{c} Ŷ=\arg\underset{j}{\max}\left(\overset{B}{\underset{b=1}{\sum }}d_{b,j}\right) \end{array}$$

Ŷ is the final predicted label using the majority voting. j and b show the available classes and tree classifiers. *d*_*b,j*_ shows the decision of each tree whether the sample belongs to class j or not.

The critical difference between the random forest and the decision tree is that the random subsets of features are generated in RF. In contrast, all the possible feature splits are considered in the decision tree (47). As the RF chooses subsets of features randomly, it ensures low correlation among the decision trees. The main parameters of RF models, like the number of trees, the maximum depth of each decision tree, minimum samples per leaf, minimum samples per split, and maximum number of features for the best split, were found using the training and validation set of data during the training process.

##### 2.3.4.4 Experiment setup

We used the 5-fold cross-validation methods for all the above classifiers to divide the available data to train and test data. To find the generalized model before applying it to the test data, the train data was split into 80% for training and 20% for validation. Also, the grid search strategy was used for all the classifiers to find the optimal parameters of the models. Various quantitative metrics of accuracy, sensitivity, precision, specificity, and F-score evaluated the results of the classifiers for discrimination of MCI and HC.

## 3 Results

The demographic and clinical information of the participants in this study who completed all the gait tests is presented in Table 1. There were significant differences in BMI and years of education between MCI and HC participants, but no differences in age or gender. Participants with MCI had higher levels of depression on the GDS score, with lower MMSE and MoCA than the HC group. To ensure the robustness of our findings, we adjusted the gait feature analysis for these confounding variables, including BMI, education, and GDS.

To examine the straight and oval path walking sensitivity for detecting MCI, we extracted a comprehensive collection of 50 features. This collection included 2 macro, 24 micro temporal, 18 micro spatial, and 6 micro spatiotemporal features. Table 2 shows the extracted gait features for gait tests in different paths. The values of the gait features are presented as the mean, standard deviation, and the *p*-values of each gait feature for the comparison between the study groups.

### 3.1 Feature selection outcome

#### 3.1.1 Statistical approach

##### 3.1.1.1 Significant gait features

Our comparative analysis of extracted gait features demonstrated significant differences between MCI and HC participants, which were more pronounced in oval path walking than in straight walking. Specifically, in the oval path conditions, 27 out of 50 extracted features showed significant differences, whereas, in straight walking, only 20 features were significant. Notably, the significant features in oval walking included two macro features (average velocity and cadence) and various micro gait features 12 temporal, 9 spatial, and 4 spatiotemporal. In contrast, straight walking yielded significant results primarily in micro gait features 5 temporal, 13 spatial, and 2 spatiotemporal.

**Figure 4:**
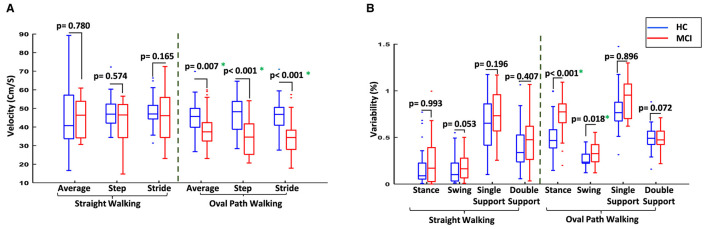
Comparison of the gait between two study groups in different conditions. **(A)** Comparison of average velocity and the velocity of steps and strides for MCI vs. HC. **(B)** Comparison of the variability of gait cycle subphases for MCI vs. HC. ^*^Significant difference at *p*-value < 0.05.

Macro features such as average velocity and cadence significantly differed in oval path walking, with MCI participants exhibiting lower average speed (39.27 ± 10.09 cm/s) and cadence (55.00 ± 11.67 steps/min) compared to HC participants (45.90 ± 8.89 cm/s and 62.02 ± 9.73 steps/min), with *p*-values of 0.007 and 0.018, respectively. Similar changes were observed for step and stride velocity between MCI and HC when the walking test changed from straight to oval path walking, and only the significantly lower step and stride velocity for MCI than HC were observed in oval path walking. This suggests a greater impact of the walking path shape on gait dynamics in MCI patients. Figure 4A compares the average velocity and the step and stride velocity between two study groups in straight and oval path walking conditions.

Furthermore, changes in gait cycle duration were observed when switching from straight to oval walking. For MCI participants, the gait cycle increased from 1.54 ± 0.22 seconds to 2.08 ± 0.53 seconds (*p*-value = 0.016), compared to a more stable change from 1.55 ± 0.30 seconds to 1.77 ± 0.26 seconds among HC participants (*p*-value = 0.254). Examining the subphases of the gait cycles provided more information on the participant's gait for comparison. Older adults with MCI generally had more variability for the subphases of the gait cycles, such as stance and swing time and the single and double support time. The differences between the variability of these subphases increased in oval walking, and even significant differences were observed for stance and swing times in oval path walking between MCI and HC participants (Figure 4B). This indicates a significant deterioration in gait coordination for MCI participants under more challenging walking conditions.

##### 3.1.1.2 Selected gait features

**Figure 5:**
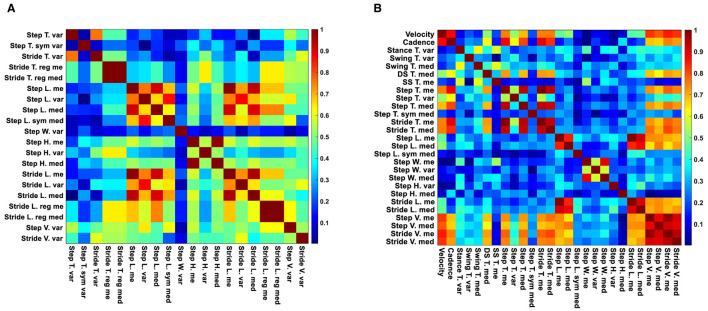
Correlation heatmaps of significant features for straight and oval path walking. **(A)** Correlation heatmap of significant features for MCI vs. HC during straight walking. **(B)** Correlation heatmap of significant features for MCI vs. HC during oval path walking.

Following our feature selection algorithm, we focused on a smaller set of unique gait features that showed the most substantial differences between the two study groups without high inter-feature correlation. Figure 5 illustrates this with a heatmap of correlation matrices for significant features from straight and oval path walking. This visualization confirms significant correlations among certain features, guiding our selection towards those with the lowest *p*-values, indicative of pronounced differences between MCI and HC participants.

Ultimately, from the oval path walking test, we selected 19 out of 27 significant features, including two macro (average velocity and cadence), 8 micro temporal (mean of single and double support time, the median of stance and swing time, and stride time, the variability of stance and swing time, and the median of the step time symmetry), 7 micro spatial (mean of step length and width, the median of step and stride length and the symmetry of step length, and the variability of step height and width), and 2 micro spatiotemporal features (mean of step velocity and the median of stride velocity).

From straight walking, 13 out of 20 significant features were retained, including 3 micro temporal (mean and median of stride time regularity and the variability of step time symmetry), 8 micro spatial (mean of step height and stride length regularity, median of step length and stride length regularity, and the variability of step length, width, and height, and the stride length), and 2 micro spatiotemporal features (variability of step and stride velocity).

For the straight walking, the selected feature consisted of 3 micro temporal features (mean and median of stride time regularity and the variability of step time symmetry), 8 micro spatial features (mean of step height and stride length regularity, median of step length and stride length regularity, and the variability of step length, width, and height, and the stride length), and two micro spatiotemporal features (variability of step and stride velocity). Table 3 summarizes the number of different types of extracted, significant, and selected features for straight and oval path walking.

**Table 3:** The numbers of various types of extracted, significant, and selected gait features using the statistical and ML approaches in both conditions.

**Type of feature**	**Extracted**	**Statistical approach**				**RF approach**	
		**Significant**		**Selected**		**Straight**	**Oval**
		**Straight**	**Oval**	**Straight**	**Oval**		
Macro	2	0	2	0	2	0	1
Micro temporal	24	5	12	3	8	3	7
Micro spatial	18	13	9	8	7	5	4
Micro spatiotemporal	6	2	4	2	2	2	4
**Total**	**50**	**20**	**27**	**13**	**19**	**10**	**16**

RF, random forest.

#### 3.1.2 Machine learning approach

**Figure 6:**
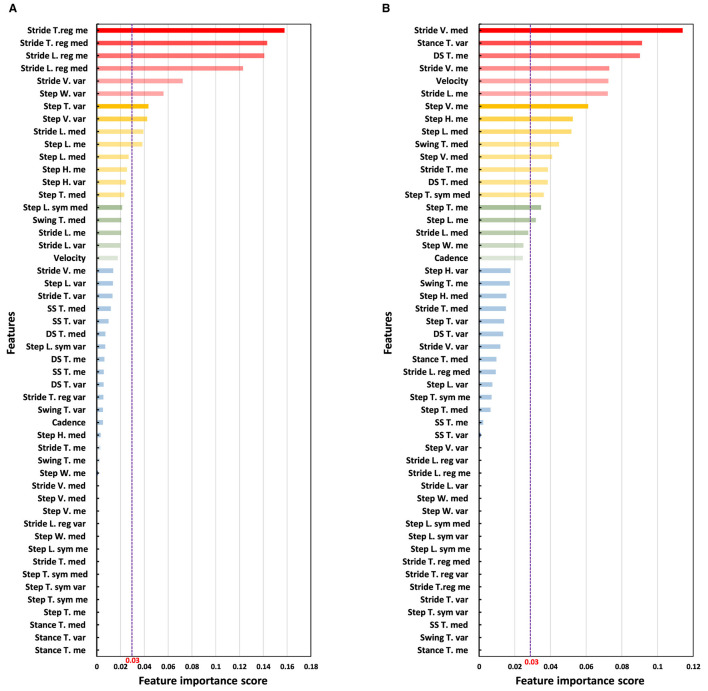
Analysis of the importance of features using the RF classifier. The purple dash line shows the threshold of 0.03 for the scores of the importance of the features provided by RF classifier. **(A)** Ranking the importance of straight walking features for discrimination of MCI and HC. **(B)** Ranking the importance of oval path walking features for discrimination of MCI and HC.

We employed the RF algorithm to assess the importance of gait features from straight and oval path walking conditions, each analyzed separately, to distinguish between MCI and HC participants. Figure 6 displays the results from the RF classifier, highlighting the importance scores of gait features under both walking conditions. As illustrated in Figure 6, the features garnered higher importance scores predominantly identified by our statistical approach, which utilized descriptive statistics and correlation analysis for feature selection. For instance, in straight walking (Figure 6A), the five features with the highest importance scores were the mean and median of stride time and length and variability of stride velocity. These features correspond to those selected through our proposed feature selection algorithm. Similarly, in oval walking, the top-ranked features by RF, such as the median of stride velocity, variability of stance time, mean of double support time, stride velocity, and average velocity (Figure 6B), aligned closely with those identified through our feature selection method. Table 3 lists the features with an RF importance score greater than 0.03.

### 3.2 MCI detection using machine learning

We designed three different classifiers to discriminate the HC and MCI participants using selected features of straight and oval paths walking separately. It is important to note that for the SVM and LR classifiers, our *statistical feature selection* method was applied within a 5-fold cross-validation framework. This means that feature selection was conducted separately on the training data of each fold, ensuring that only the features identified from the training data were used to train the models, thereby preventing information leakage to the test data. We used the entire set of features for the RF classifier, allowing the algorithm to autonomously select the most predictive features within each cross-validation fold. We chose 5-fold cross-validation because it balances computational efficiency and validation accuracy (48). This approach is optimal for our dataset size, reducing the risk of overfitting and variance compared to 10-fold or leave-one-out methods, which can be computationally intensive and less stable for moderate sample sizes.

As seen in Table 4, the classification accuracy of participants to MCI and HC groups using straight walking features were 65.8%, 76.1%, and 78.2% for LR, SVM, and RF, respectively. Also, the F-scores were 61.7%, 72.6%, and 75.9% for those classifiers in the same walking condition. In comparison, the results generally improved in oval path walking. The classification accuracy increased to 74.2%, 80.1%, and 85.50% for LR, SVM, and RF, respectively, when the features of oval path walking were used. Similar improvements were also seen for F-scores and other evaluation metrics, where the F-scores of these classifiers rose to 72.4%, 77.9%, and 83.9%. The comparison of the classification results using different classifiers and gait tests showed that the RF classifier on the oval path walking data had the best performance for discrimination of MCI and HC with accuracy and F-score of 85.5% and 83.9%. The other evaluation metrics, such as sensitivity, precision, and specificity, confirmed this finding, too.

**Table 4:** Classification results for different types of classifiers and gait tests.

**Gait test**	**Classifier**	**Evaluation metrics (%)**				
		**Accuracy**	**Sensitivity**	**Precision**	**Specificity**	**F-score**
Straight	LR	65.8	60.0	63.6	71.7	61.7
	SVM	76.1	68.0	78.2	84.2	72.6
	RF	78.2	73.0	79.2	83.3	75.9
Oval	LR	74.2	75.0	70.1	73.3	72.4
	SVM	80.1	76.0	80.2	84.2	77.9
	RF	85.5	81.0	87.1	90.0	83.9

LR, logistic regression; SVM, support vector machine; RF, random forest.

## 4 Discussion

Early detection of AD and dementia is pivotal in slowing or potentially preventing their progression to more severe stages, especially given the current lack of a cure for these degenerative diseases. Identifying MCI, a key precursor to AD, is therefore crucial. Individuals with MCI convert to AD at a higher annual rate than their cognitively healthy counterparts, as highlighted in Shigemizu et al. (7). Conventional clinical methods for MCI detection, including neuropsychological tests, brain imaging, EEG, and blood tests, are often time-consuming and costly, and the practitioner's experience can influence their effectiveness. To address these limitations, our study introduces a novel method for MCI detection employing comprehensive gait analysis during both oval and straight walking patterns, captured using a Kinect v.2 depth camera. This approach integrates signal processing, descriptive statistical tools, and machine learning techniques. Our method presents an objective, non-invasive, and easy-to-implement alternative for MCI detection, offering a low-cost and less time-intensive solution suitable for both clinical and non-clinical settings. Data was collected from 55 older adults, comprising 25 individuals with MCI and 30 HC, to validate this approach.

### 4.1 Main findings and implications

The study highlighted significant gait performance differences between MCI and HC groups. Older adults with MCI showed weaker performance, especially in oval path walking, resulting in more noticeable differences between MCI and HC groups than straight walking (Table 2). In oval path walking, 27 out of 50 features showed significant differences between MCI and HC, compared to 20 features in straight walking (Table 3). Older adults with MCI demonstrated significantly lower average velocity and cadence during oval walking, while these macro gait features showed no significant differences in straight walking (Figure 4A). Similar trends were observed for step and stride velocity, with significant differences in oval walking but not straight walking conditions (Figure 4A). Analyzing gait subphases revealed increased variability in stance, swing, single support, and double support among MCI individuals, especially during oval walking, resulting in significant differences in stance and swing time between MCI and HC (Figure 4B). These results are backed by previous research in the clinical field, showing that various factors contribute to these outcomes. When walking along curves like an oval path, individuals with MCI face heightened cognitive demands, necessitating additional time to adapt to changes in direction and maintain their balance (49, 50). Additionally, they exhibit more cautious movement along oval paths due to increased worries about falling, difficulties in spatial awareness, and intricate motor planning (51, 52). Our findings and this clinical evidence emphasize how gait features can detect the difficulties presented by various types of curved walking around an oval path and their potential role in indicating cognitive impairment. Also, our findings align with previous clinical research, which reported no significant differences in average velocity and cadence during straight walking between MCI and HC (53, 54). Despite a lack of comprehensive studies comparing oval and straight walking tests between MCI and HC, previous research analyzing other curved walking aspects, such as the turning part of the TUG test, showed significant differences between MCI and HC (19, 55). Our study extends these findings, highlighting the higher sensitivity of oval walking compared to straight walking in detecting MCI, providing a detailed gait analysis over a longer curved path than previous studies.

Another main observation was that we identified optimal gait features with enhanced sensitivity in distinguishing between MCI and HC through a two-step feature selection algorithm involving descriptive statistics and correlation analysis. This process yielded a reduced set of gait features suitable for efficient MCI detection in both oval and straight walking tests, with 19 features for oval walking and 13 for straight walking (Figure 5 and Table 3). These selected features serve as focal points for clinicians, streamlining MCI detection and expediting machine learning procedures by eliminating redundant features. Further validation using the RF classifier corroborated the effectiveness of our feature selection methods. RF highlighted the most influential features for MCI detection, aligning with those identified through our selection algorithms (Figure 6).

Furthermore, various classifier models were employed to detect MCI in different oval and straight walking conditions, highlighting the higher sensitivity of oval walking for MCI detection. The Random Forest model demonstrated superior performance among the classifiers. In oval walking, RF achieved an accuracy and F-score of 85.5% and 83.9%, while in straight walking, these metrics were slightly lower at 78.2% and 75.9% (Table 4). RF's enhanced performance can be attributed to its ensemble learning method, which combines multiple decision trees and aggregates their predictions, thereby reducing overfitting and enhancing model diversity (5, 47, 56, 57).

### 4.2 Comparison with previous studies

Our literature review revealed a scarcity of studies examining detailed gait analysis in oval and straight walking conditions using sensor technology and machine learning methods. However, several studies have developed systems to analyze the gait of older adults with cognitive impairment using machine learning for disease assessment (Supplementary Table S2, Supplementary Section S7).

In the context of AD versus HC detection, which typically shows higher performance due to more pronounced differences, Wang et al. (58) used inertial-sensor-based devices to record the gait of 30 AD and 30 HC participants. They achieved a 66.7% accuracy in distinguishing between the two groups using probabilistic neural networks, without considering gender differences (58). Varatharajan et al. further demonstrated this with wearable sensors and dynamic time-warping methods, classifying 150 HC and 173 AD older adults with an accuracy of 94.5% and a sensitivity of 95.9% (59). Zhang et al. (60) combined the skeletal map of single and dual-task gait recorded with a Kinect camera and fed to a Convolutional Neural Network (CNN) to classify older adult participants (HC = 106, Dementia = 194) to older adults with dementia and without dementia. Their developed methods used the features extracted using the CNN instead of the feature engineering methods to extract the features and then feed them to the classifier. They reported a sensitivity 74.1% for detecting older adults with dementia (60). Seifallahi et al. (61) suggested a comprehensive analysis of the Timed Up and Go test (TUG) via recorded data with a Kinect v.2 camera combined with signal processing and machine learning for the detection of AD at the mild to moderate stages while the overall duration of TUG test commonly is measured at the clinic with a stopwatch. Their suggested methods provided more detail about the performance of older adults with mild to moderate AD compared to HC older adults and reported the discrimination of 38 AD and 47 HC participants with accuracy and F-scores of 97.7% and 97.7%, respectively (61).

MCI detection, which involves subtler gait differences than AD, presents greater challenges. Gwak et al. (21) proposed a gait measurement system using a smartwatch, employing processing algorithms based on two gait features and statistical features. They achieved 88.0% accuracy for distinguishing between 27 individuals with MCI and 26 HC participants using logistic regression (21). Ghoraani et al. (22) analyzed gait in single and dual-task conditions using an electronic walkway, extracting features for 32 HC vs. 26 MCI and 20 AD older adults. Applying SVM, they reported an average accuracy and F-score of 88.0% and 90.0% for HC vs. MCI/AD classification (22). Shahzad et al. (62) proposed recording the 10-meter straight walking in single and dual cognitive tasks of counting backward from 70 by 1's and speaking out animal names with a single wearable sensor of Shimmer mounted on the mid-shank of each subject. Their analysis using descriptive statistical analysis for comparison between two study groups (MCI = 30 and HC = 30) revealed a significant difference for all gait tests from single to dual cognitive tasks, even though more numbers of significant features were observed for the dual cognitive task of naming animals during 10-meter straight walking. They used various feature engineering classifiers, including the DT, RF, and ANN, with different feature selection algorithms like correlation and mutual information to automatically detect MCI. They reported the highest performance based on the sensitivity of MCI detection with the sensitivity of 83.3% and accuracy of 71.7% for the SVM and the mutual information feature selection algorithm (62). More recently, Jeon et al. (63) utilized wearable sensors for gait measurement in 68 MCI and 77 HC older adults during straight walking. Their proposed ensemble algorithm showed improved MCI detection with a 73.0% accuracy (63). Russo et al. (8) recorded the straight walking using an optical system equipped with six IR cameras, two video cameras, two force plates, and a set of 26 passive reflective markers mounted on the participant's body to discriminate 40 PD patients with MCI and without MCI. The participants performed the single straight 10 meters, dual motor gait with carrying a tray with two filled glasses with water, and dual cognitive gait of subtracting from 100 by 7's during walking. They extracted 16 Spatio-temporal gait features from each of the different types of gait tests (48 gait features in total). After finding significant gait features and applying the feature selection, various ML feature engineering models consisting of RF, KNN, NB, and DT were applied to the extracted features, and they reported the highest accuracy of over 80% via SVM and RF for the classification of participants to two study groups as PD-MCI and PD-No MCI. Also, they used the Wrapper method to find the selected features with the most power to discriminate PD-MCI and PD-No MCI and reported 17 selected features while the majority of them belonged to dual cognitive gait (8)

Our study contributes to the growing body of research on gait analysis and machine learning for MCI detection, resonating with findings from prior studies (8, 21, 22, 58–63). Unlike earlier research that primarily relied on wearable sensors or complicated and expensive systems made up of several IR cameras and reflective markers on the participant's body, which might influence gait patterns and suffer from signal inconsistencies due to sensor movement or user-induced changes (8, 21, 58, 59, 62, 63), our approach utilized a single Kinect v.2 camera. This non-wearable device enables an unobtrusive setup, providing comprehensive data from 25 body joints without impacting natural gait.

Furthermore, while some studies employed electronic walkways that faced limitations in space requirements, setup complexity, and specific gait feature extraction (22), our method overcomes these challenges by capturing a wider range of gait features, including step and stride height. Our focus on MCI detection, a subtler and more challenging transitional stage to ADRD, also sets our study apart from those mainly targeting dementia detection (58–61). The size of our study population was comparable to that of Gwak et al. (21), Ghoraani et al. (22), and Shahzad et al. (62), but smaller than the cohort in Jeon et al. (63). Like these studies (21, 22, 63), we tackled the intricate task of detecting less pronounced gait defects in MCI, compared to studies focusing on AD and HC (58, 59, 61).

Moreover, our study uniquely contributes by directly comparing the sensitivity of gait detection between oval and straight walking patterns for MCI detection. This specific comparison has not been thoroughly examined in existing literature, distinguishing our research in its approach to understanding how different walking paths may influence the detection of MCI. Previous studies only examined the straight walking for MCI, AD, or ADRD detection (8, 21, 22, 58–60, 62, 63) or a short duration of turning of the TUG test for AD detection (61). Oval walking, more reflective of daily activities, demonstrated higher sensitivity in identifying MCI. Moreover, we pinpointed a smaller, more effective set of gait features, enhancing the efficiency of MCI detection a finding not fully explored in previous studies (22).

In terms of machine learning performance, our Random Forest model achieved 85.50% accuracy and 83.9% F-score in oval walking for MCI detection, on par with some previous studies (21, 22), but outperforming others (62, 63). Notably, our analysis focused solely on single-task oval walking and accounted for confounding factors, unlike Ghoraani et al., who combined single and dual-task walking data (22), and Gwak et al., who did not adjust for confounders (21). These distinctions underscore the potential of single-task oval walking for MCI detection, warranting further investigation.

### 4.3 Clinical implications

Our study marks a pivotal advancement in the screening of MCI. Utilizing a Kinect v.2 camera, we have developed a non-invasive, cost-effective, and efficient method to analyze gait patterns in both oval and straight walking tests. This technique stands out for its practicality in clinical settings, where it can serve as an initial screening tool for MCI. Its primary advantage is detecting subtle gait irregularities, often early indicators of cognitive decline, that may not be evident in standard clinical evaluations. From a clinical perspective, this approach could significantly streamline the early detection process of MCI, facilitating prompt intervention. Integrating this method into standard geriatric assessments could revolutionize the early detection process for MCI, enabling healthcare professionals to swiftly identify and subsequently guide at-risk individuals toward comprehensive cognitive assessments and timely intervention.

### 4.4 Study limitations and future work

The sample size of this study, while consistent with similar research in the field (see Supplementary Table S2), is relatively small, with a high feature-to-sample size ratio, which may limit the generalizability of our findings and heighten the risk of overfitting. Our future studies aim to include larger cohorts to validate and potentially enhance the reliability of the machine learning approaches described herein. Expanding the sample size will be crucial for confirming the efficacy of our method and its applicability to a broader population, particularly for clinical applications in detecting MCI among diverse groups. Our MCI diagnoses were based on clinical criteria, rather than biomarker confirmation so it is unknown if the MCI group in this study had AD as the underlying etiology. Similarly, the HC group could be contaminated with preclinical AD cases. Future studies should include biomarker confirmed groups.

Additionally, we will explore a wider array of gait and balance evaluations, such as the Timed Up and Go (TUG) test, to refine our screening methodology further. In line with technological advancements, we are also considering the integration of regular cameras, combined with deep neural network models, for gait analysis. This potential enhancement could make our method more versatile and accessible, suitable for various clinical and non-clinical settings. These future initiatives are anticipated to augment the effectiveness of our current approach and make substantial contributions to the fields of geriatric care and neurodegenerative disease research. Ultimately, through these advancements, we aim to improve patient outcomes by facilitating earlier detection and intervention strategies for neurodegenerative diseases like MCI and ADRD.

While the Kinect v.2 camera is sensitive to environmental conditions like lighting and has a limited operational range of 0.5 to 4.5 meters, it remains a practical choice for gait analysis due to its affordability, ease of use, and non-invasive tracking of multiple joints without wearable sensors. These features are particularly advantageous for studies involving older adults or individuals with cognitive impairments. Acknowledging its limitations, we plan to enhance our research methodology by integrating newer technologies, such as the Azure Kinect, which offers improved depth sensing and tracking capabilities (see Supplementary Section S1). Additionally, exploring hybrid systems that combine Kinect's capabilities with high-precision motion capture technologies will aim to overcome current limitations and refine the accuracy of our gait analysis. These future directions are geared toward developing more reliable diagnostic tools for cognitive impairments.

## 5 Conclusion

This study represents a significant stride forward in early ADRD detection during the MCI stage. We have introduced a novel, cost-effective tool leveraging a single Kinect v.2 camera to accurately track 25 body joints during both oval and straight walking patterns. Our comprehensive approach, which combines advanced signal processing, meticulous statistical analysis, and sophisticated machine learning techniques, facilitates the extraction of critical gait features. These features are then adeptly analyzed by machine learning models, particularly focusing on MCI detection. Our findings reveal a notable sensitivity in oval walking, where 27 significant gait features were identified in distinguishing MCI from HC, compared to 20 in straight walking. The Random Forest classifier demonstrated exceptional performance in analyzing oval walking gait measurements, achieving a notable 85.5% accuracy and an 83.9% F-score in detecting MCI. Furthermore, aligning the most important features identified by the Random Forest classifier with those selected through our feature selection methods suggests the potential to refine our model to focus on a smaller yet more effective set of gait characteristics for early ADRD detection. This study underscores the viability of using the Kinect v.2 camera and gait analysis as a powerful tool for early ADRD detection at the MCI stage. Characterized by its affordability, efficiency, simple setup, and non-invasiveness, this method is highly suitable for clinical and non-clinical environments. Its use in routine gait screening could significantly advance early ADRD detection, enabling timely interventions and potentially altering the trajectory of cognitive decline. Our research opens new avenues in geriatric care and neurodegenerative disease management, marking a shift in approaching early AD detection and prevention.

## Data availability statement

The raw data supporting the conclusions of this article will be made available by the authors, without undue reservation.

## Ethics statement

The studies involving humans were approved by the Ethics Committee of Semnan University of Medical Sciences of Iran. The studies were conducted in accordance with the local legislation and institutional requirements. The participants provided their written informed consent to participate in this study.

## Author contributions

MS: Conceptualization, Formal analysis, Methodology, Writing – original draft, Data curation, Investigation, Software, Validation, Visualization. JG: Validation, Writing – review & editing, Conceptualization, Investigation, Methodology. BG: Conceptualization, Formal analysis, Funding acquisition, Methodology, Supervision, Writing – review & editing, Investigation, Project administration, Resources, Validation.
