# Supplementary material for: Detection of mild cognitive impairment using various types of gait tests and machine learning
Source: Front Neurol. 2024 Jul 11;15:1354092. doi: 10.3389/fneur.2024.1354092 (PMC11269186; doi:10.3389/fneur.2024.1354092)
Supplement: Supplementary file 1 [file Data_Sheet_1.pdf]

Supplementary Material

S1 COMPARISON OF KINECT V2 WITH AZURE KINECT

Table S1 highlights the key features, operational capabilities, and limitations of the Kinect v.2 and Azure Kinect systems used in gait analysis. This table provides a succinct overview of why Kinect v.2 was chosen for our study, considering its cost-effectiveness and proven utility for motion tracking in clinical and non-clinical settings. It also outlines potential future enhancements using newer technologies like the Azure Kinect, which offers improved resolution and depth sensing.

S2 OVAL PATH WALKING

Figure S1 shows the details of the walking on the oval path. The oval path walking path had a major and minor radius with a length of 2 and 1 meters, respectively. The angle degree of the oval circle was 45°. The meaning of the angle degree here is that this oval path is like rotating a circle for 45° to become an ellipse at that angle of view.

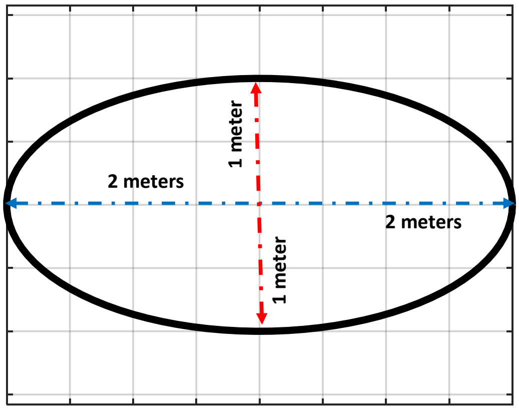

Figure S1: Oval path walking with the detail of its shape

Table S1. Comparison of Kinect v.2 and Azure Kinect in Movement Acquisition.

Features	Kinect v.2	Azure Kinect
**Release Year**	2014	2020
**Operation Principle**	ToF with modulation up to 130 MHz	ToF with modulation 200–300 MHz
**Sensors and Data Types**	RGB camera, IR sensor, and IR projector producing RGB images, depth images, and skeletal data from 25 body joints.	RGB camera, Depth camera, IR emitter, Gyroscope and accelerometer producing RGB images, depth images, and skeletal data from 32 body joints
**Camera Specifications**	RGB: 1920x1080 pixels at 30 fps, FoV of 84.1° x 53.8°. IR: 512x424 pixels at 30 fps, FoV of 70.6° x 60°.	RGB: 3840x2160 pixels at 30 fps, FoV of 90° x 59°. IR: Various modes (WFOV: 1024 × 1024 px, NFOV: 640 × 576 px)
**Operational Range**	Effective within a 0.5 to 4.5 meters range. It can track up to six individuals simultaneously.	It has a different operational range based on the depth camera mode (WFOV: 0.25 to 2.88 meters, NFOV: 0.50 to 5.46 meters). It can track up to six individuals simultaneously.
**Cost and Accessibility**	Affordable and widely accessible, making it suitable for domestic and clinical settings.	More expensive due to higher specifications and requires more powerful hardware for operation.
**Limitations**	Sensitive to lighting conditions and has a limited tracking range. Direct sunlight can impair tracking accuracy.	While it has fewer environmental limitations, such as less sensitivity to sunlight and temperatures, the higher cost and hardware requirements could limit accessibility.
**Mitigation Strategies**	Optimal positioning, interpolation for self-occlusion, controlled environment, and repeat recordings to ensure accuracy.	Similar strategies to manage environmental and operational challenges.
**Comparative Advantage**	Proven reliability and validity in gait analysis. Favorably compared to systems like GaitRite.	Promises higher accuracy and resolution but requires validation for specific applications like gait analysis in older adults.

ToF= Time-of-flight; *IR* = Infrared; *FoV* = Field of view; *fps* = Frame per second; *WFOV* = Wide field of view; *NFOV* = Narrow field of view.

S3 OPEN-SOURCE TOOLS AND SOFTWARE PACKAGES FOR DATA ACQUISITION USING KINECT V.2 AND ANALYSIS

For data acquisition with Kinect v.2, we utilized the SDK provided by Microsoft, which is well-documented and publicly accessible. Additionally, we employed several MATLAB

packages for signal processing, feature extraction, and classification, which are integral to our analysis framework:

- **Kinect for Windows SDK:** Kinect for Windows SDK
- **Acquire Image and Body Data:** MATLAB support for Kinect v.2
- **View Skeletal Data:** Viewing Skeletal Data with MATLAB
- **Acquire Data from Kinect Color and Depth Devices Simultaneously:** MATLAB Documentation
- **Signal Peak Analysis:** MATLAB findpeaks function
- **Support Vector Machine Classification:** MATLAB SVM Classification

S4 METHODOLOGY AND ENVIRONMENTAL SETUP FOR GAIT ANALYSIS USING KINECT V2

Our literature review and subsequent analyses corroborate findings from studies such as those by Galna et al. (2014) and Gonzalez-Jorge et al. (2015), which document the spatial and temporal accuracy of Kinect v2. While the spatial accuracy for static postures was superior, dynamic activities like walking demonstrated increased errors with greater distance from the camera, which is crucial for gait analysis. We designed our data recording setup to address these concerns and optimize accuracy and reliability.

- **Controlled Clinical Environment:** Data was recorded in a controlled clinic environment to ensure no external occlusion with other people. This setup helped minimize interference and maximize the clarity of the recorded data, which is essential for accurate joint tracking and gait analysis.
- **Lighting and Sunlight Control:** The recording room was equipped with curtains to counteract the effects of severe sunlight, which could affect the camera's sensitivity. Additionally, the room lighting was carefully managed with sufficient luminance from light lamps to ensure consistent lighting conditions, which is crucial for maintaining the accuracy of the Kinect's depth-sensing capabilities.
- **Optimal Camera Placement and Environment Control:** We positioned the Kinect v2 camera primarily in the Anterior-Posterior (AP) direction for straight walking paths to minimize occlusion and enhance the accuracy of gait marker measurements. This setup helps reduce errors associated with lateral movements and body self-occlusions, as Otte et al. (2016) and Cai et al. (2021) recommended. For the oval path, the camera was positioned laterally to accommodate the limited space available in the clinic and to capture the gait from a side perspective. This setup also reduced potential self-occlusion

issues that were more prevalent in lateral movements. It is important to note that healthy controls and MCI participants underwent gait analysis under identical camera setups for straight and oval path walking tests. This ensured that any differences observed in detecting MCI were due to genuine gait variations and not influenced by differences in camera placement. The consistency in setup across all tests supports the reliability of our findings, confirming that the increased sensitivity for MCI detection on oval paths reflects true differences in motor control.

- **Focus on Lower Body Joints and Interpolation for Missing Data:** Recognizing the reliability of Kinect v2 in tracking lower body joints, our analysis concentrated on these areas. For occlusion or incomplete data, we utilized cubic spline interpolation to estimate the positions of occluded joints, ensuring continuity and accuracy in our data set.
- **Participant Tracking and Data Consistency:** We took special measures to prevent any changes in participants' identification (ID) during the walking tests. The path and camera were arranged to keep the participant in the camera's view continuously, ensuring consistent tracking throughout the test. For instances of self-occlusion, particularly in the oval path walking where the Kinect v2 camera was in the side view, the untracked joint was estimated using cubic spline interpolation, a method supported by previous studies (Capecci et al., 2016).
- **Utilization of Confidence Scores:** We incorporated Kinect's JointTrackingStates, which provide a confidence score for each joint detected (0 for not tracked, 1 for inferred, and 2 for tracked). This helped us refine our data handling, where joints with a confidence score of 0 were particularly subjected to interpolation methods to estimate their likely positions.

By managing these aspects meticulously, we leveraged Kinect v2 effectively for rigorous gait analysis despite its known limitations in dynamic environments.

S5 DATA DISPLAY FOR STRAIGHT AND OVAL PATH WALKING

Our analysis of the recorded data from Kinect v.2 shows that the Kinect camera can detect and track the 25 joints of the body. At the same time, its accuracy can slightly change regarding issues such as the camera's distance from the person. Our finding generally shows that this error was by 2 cm, reported in previous studies too Gonzalez-Jorge et al. (2015). Figure S2 shows the analysis of the skeletal data while performing the straight and oval path walking, and the person's height was calculated based on the skeletal data for various frames.

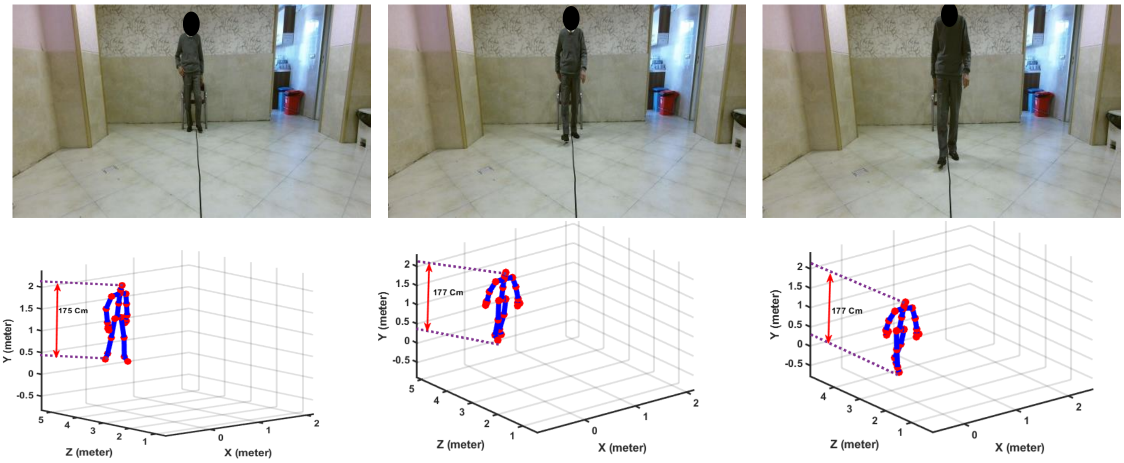

Figure S2.a: Sample recorded RGB and skeletal data during straight walking.

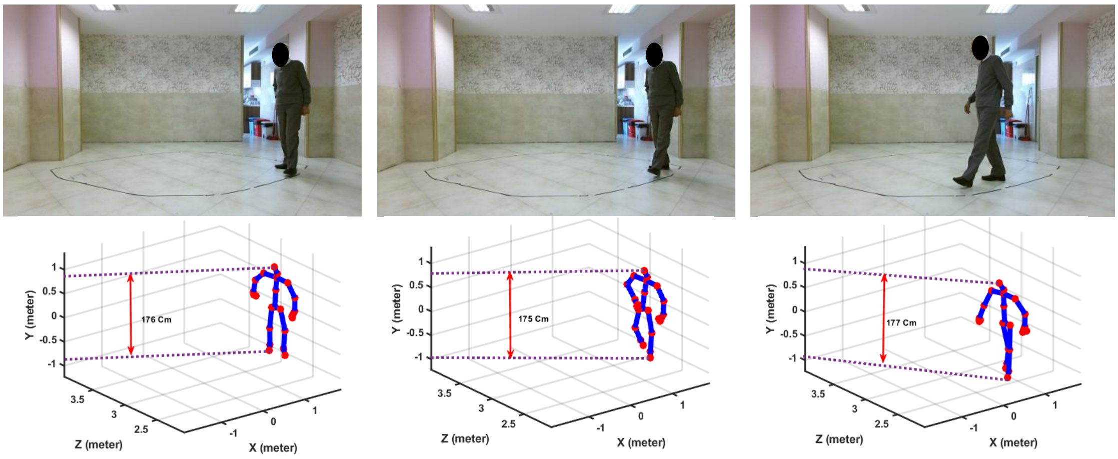

Figure S2.b: Sample recorded RGB and skeletal data during oval path walking.

Figure S2: The sample recorded data using Kinect v.2. **(A)** RGB and skeletal data for straight path walking. **(B)** RGB and skeletal data for oval path walking.

S6 ADDITIONAL ANALYSIS ON CAMERA VIEW AND GAIT DATA ACCURACY

The data were recorded in a clinical setting where clients attended their regular checkups and followed their medical care. The room had limited space for recording both tests in

the same view. The larger diameter of the oval path was 4 meters, requiring sufficient distance from the camera to track body joints accurately while turning the corner. If the path was designed in the frontal view of the camera, we would miss tracking the body joints for critical parts of the oval-path walking, as Kinect v.2 tracks body joints within a range of 0.5-4.5 meters. Placing the camera 1 meter away from the end of the walking path in a frontal view would result in missing essential sections of the oval path walking. Conversely, positioning the camera in the sagittal view for the oval path allowed us to record the entire path. However, it introduced challenges such as partial self-occlusion, which we addressed with interpolation methods detailed in supplementary section S4. If the oval path had a shorter main diameter, it would not meet the standard 10-meter walking requirement, compromising the typical walking pattern of participants. Thus, we made a trade-off between the camera view and the potential decrease in accuracy for tracking body joints. We believe that while the change in camera view may affect signal accuracy from the body joints and extracted gait features, both study groups (HC and MCI) would be similarly impacted, ensuring the observed trends in gait features and results comparisons remain valid.

To evaluate the effect of camera view changes and other factors (e.g., walking speed, Kinect v.2 camera temperature, participants' clothing) on measured body joints, we performed further analyses on the recorded data. Since the distance between ankle and foot joints should remain consistent during frames, we measured this distance while walking on straight and oval paths for each subject, focusing on these joints as many extracted features rely on their signals. We calculated the mean and standard error of the ankle-foot distance during recorded frames to assess variation. Our analysis revealed a tolerance for estimated body joints by the Kinect v.2 in both straight and oval paths (frontal and sagittal views). In the frontal view, the ankle-foot distance was measured using Z-axis signals, whereas in the sagittal view, it was based on X-axis signals. Figure S3 illustrates the mean ankle-foot distance and standard error relative to the mean values for recorded frames in both study groups. The error range was slightly greater in oval-path walking (sagittal view), potentially due to higher accuracy in the frontal view. To determine if this difference was significant, we conducted paired t-tests on estimated ankle-foot distances for participants, comparing frontal and sagittal views. The analysis yielded no significant differences, with p-values of 0.5032 and 0.6938 for the HC and MCI groups, respectively.

Additionally, we compared the changes in estimated ankle-foot distances between frontal and sagittal views for MCI and HC participants to verify if camera view changes similarly affected both groups. Figure S2 presents a boxplot of the absolute changes in estimated ankle-foot distances, and an unpaired t-test reported a p-value of 0.7010, indicating no

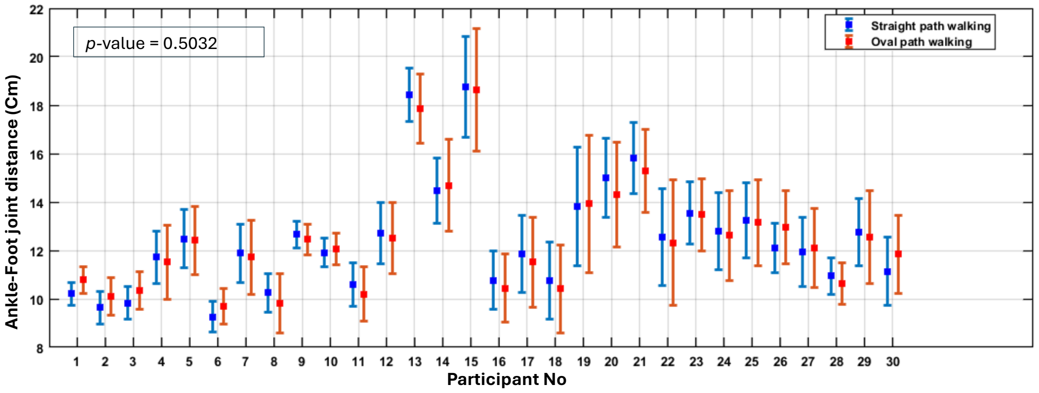

Figure S3.a: Analysis of HC participants

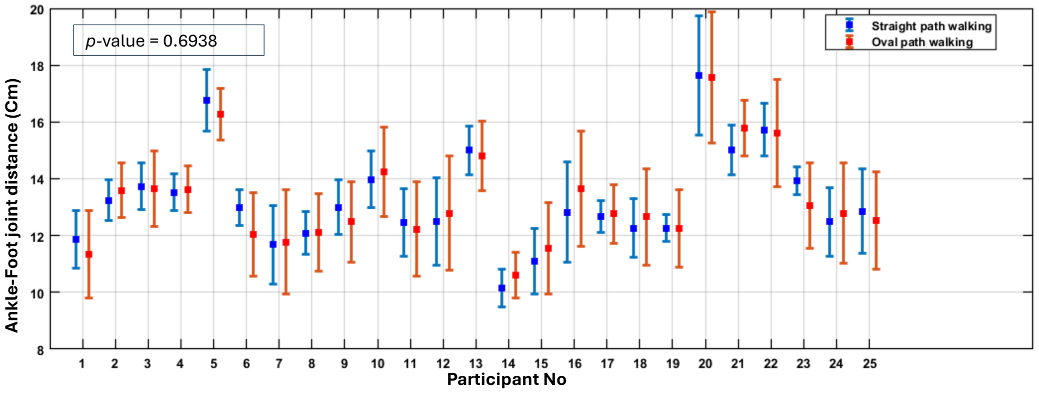

Figure S3.b: Analysis of MCI participants.

Figure S3: Comparison of Ankle-Foot Joint Distance in straight (frontal view) and oval path walking (sagittal view) for HC and MCI participants. **(A)** HC participants: Mean ankle-foot joint distance (cm) with standard error bars for each participant in straight (blue) and oval path (red) walking. p-value: 0.5032. **(B)** MCI participants: Mean ankle-foot joint distance (cm) with standard error bars for each participant in straight (blue) and oval path (red) walking. p-value: 0.6938.

significant differences between the two groups. These results confirm that changes in camera view affected both groups similarly. Consequently, the variations in camera view do not impact the number of significant gait features identified in oval and straight walking

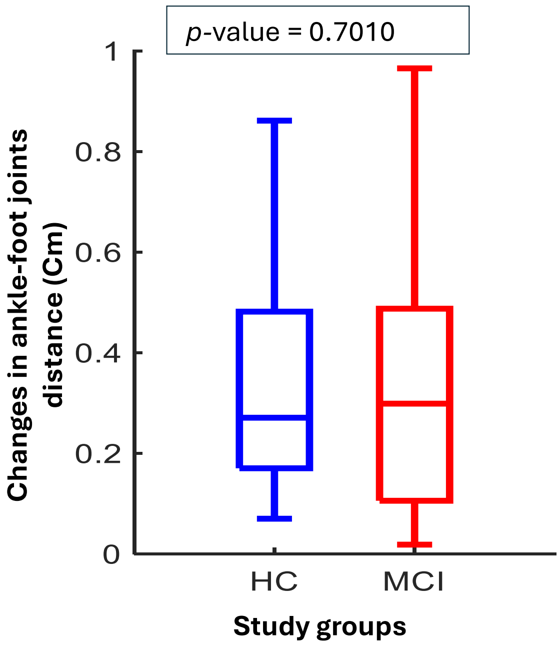

Figure S4: Effect of Camera View Change on Ankle-Foot Joint Distance Estimation for HC and MCI Participants. The boxplot shows the absolute changes in ankle-foot joint distance (cm) when the camera view is switched from frontal to sagittal. The p-value of 0.7010 indicates no significant difference between the HC (blue) and MCI (red) groups.

tests, despite a partial error in body joint position estimation, which may slightly alter measured gait features.

S7 COMPARISON WITH PREVIOUS STUDIES

Table S2 summarizes the previous papers that applied machine learning to the gait data for MCI or AD detection and our study. This table reports the best results of the studies.

Table S2. Comparison of our study with previous studies.

Study	Apparatus	Type of gait test	Population	ML model	Results (%)
Wang et al. (2014)	wearable sensor	Combined ST and DT straight walkingt	AD= 30<br>HC= 30	PNN classifier	Acc=66.7
Varatharajan et al. (2018)	wearable sensor	ST straight walking	AD= 173<br>HC= 150	DTW classifier	Acc= 94.5<br>Sen= 95.9
Gwak et al. (2018)	wearable sensor	ST straight walking	MCI= 27<br>HC= 26	LR classifier	Acc= 88.0
Ghoraani et al. (2021)	Electronic walkway	Combined ST and DT straight walking	AD= 20<br>MCI= 26<br>HC= 32	SVM classifier	Acc= 88.0<br>F-score= 90.0
Zhang et al. (2021)	Kinect v.2	Combined ST and DT straight walking	Dementia= 194<br>HC= 106	CNN classifier	Sen= 74.1
Shahzad et al. (2022)	wearable sensor	ST and DT straight walking	MCI= 30<br>HC= 30	SVM classifier	Acc= 71.67<br>Sen= 83.3
Seifallahi et al. (2022)	Kinect v.2 camera	ST of TUG test	AD= 38<br>HC= 47	SVM classifier	Acc= 97.7<br>F-score= 97.7
Jeon et al. (2023)	wearable sensor	Combined ST and DT straight walking	MCI= 68<br>HC= 77	Ensemble model using elimination the single classifier with no improving the results	Acc= 73.0
Russo et al. (2023)	IR and regular video cameras, reflective markers and force plate	ST, Cognitive and Motor DT straight walking	PD-MCI= 40<br>PD-NoMCI= 40	RF and SVM classifiers	Acc = 80
**Our study**	Kinect v.2 camera	ST oval walking	MCI= 25<br>HC= 30	RF classifier	Acc= 85.5<br>F-score= 83.9

ML= machine learning; *ST* = single task; *DT* = dual task; *TUG* = Timed Up and Go Test; *AD* = Alzheimer's disease; *MCI* = mild cognitive impairment;*HC* = healthy control group without cognitive impairment; *PNN*= probabilistic neural networks; *DTW*= dynamic time warping; *LR*= logistic regression*SVM*= support vector machine ; *RF*= random forest*;* Acc = accuracy; *Sen* = sensitivity.

REFERENCES

Galna B, Barry G, Jackson D, Mhiripiri D, Olivier P, Rochester L. Accuracy of the microsoft kinect sensor for measuring movement in people with parkinson's disease. *Gait & posture* **39** (2014) 1062–1068. doi:10.1016/j.gaitpost.2014.01.008.

Gonzalez-Jorge H, Rodríguez-Gonzálvez P, Martínez-Sánchez J, González-Aguilera D, Arias P, Gesto M, et al. Metrological comparison between kinect i and kinect ii sensors. *Measurement* **70** (2015) 21–26. doi:10.1016/j.measurement.2015.03.042.

Wang WH, Hsu YL, Pai MC, Wang CH, Wang CY, Lin CW, et al. Alzheimer's disease classification based on gait information. *2014 International Joint Conference on Neural Networks (IJCNN)* (IEEE) (2014), 3251–3257. doi:10.1109/IJCNN.2014.6889762.

Varatharajan R, Manogaran G, Priyan MK, Sundarasekar R. Wearable sensor devices for early detection of alzheimer disease using dynamic time warping algorithm. *Cluster Computing* **21** (2018) 681–690. doi:10.1007/s10586-017-0977-2.

Gwak M, Woo E, Sarrafzadeh M. The role of accelerometer and gyroscope sensors in identification of mild cognitive impairment. *2018 IEEE global conference on signal and information processing (GlobalSIP)* (IEEE) (2018), 434–438. doi:10.1109/GlobalSIP.2018.8646622.

Ghoraani B, Boettcher LN, Hssayeni MD, Rosenfeld A, Tolea MI, Galvin JE. Detection of mild cognitive impairment and alzheimer's disease using dual-task gait assessments

and machine learning. *Biomedical signal processing and control* **64** (2021) 102249. doi:10.1016/j.bspc.2020.102249.

Zhang Z, Jiang Y, Cao X, Yang X, Zhu C, Li Y, et al. Deep learning based gait analysis for contactless dementia detection system from video camera. *2021 IEEE International Symposium on Circuits and Systems (ISCAS)* (IEEE) (2021), 1–5. doi:10.1109/ISCAS51556.2021.9401596.

Shahzad A, Dadlani A, Lee H, Kim K. Automated prescreening of mild cognitive impairment using shank-mounted inertial sensors based gait biomarkers. *IEEE Access* **10** (2022) 15835–15844. doi:10.1109/ACCESS.2022.3149100.

Seifallahi M, Mehraban AH, Galvin JE, Ghoraani B. Alzheimer's disease detection using comprehensive analysis of timed up and go test via kinect v. 2 camera and machine learning. *IEEE Transactions on Neural Systems and Rehabilitation Engineering* **30** (2022) 1589–1600. doi:10.1109/TNSRE.2022.3181252.

Jeon Y, Kang J, Kim BC, Lee KH, Song JI, Gwak J. Early alzheimer's disease diagnosis using wearable sensors and multilevel gait assessment: A machine learning ensemble approach. *IEEE Sensors Journal* (2023). doi:10.1109/JSEN.2023.3259034.

Russo M, Amboni M, Barone P, Pellecchia MT, Romano M, Ricciardi C, et al. Identification of a gait pattern for detecting mild cognitive impairment in parkinson's disease. *Sensors* **23** (2023) 1985. doi:10.3390/s23041985.
